# In vitro assessment of the anti-adenoviral activity of artemisinin and its derivatives

**DOI:** 10.1016/j.virusres.2024.199448

**Published:** 2024-08-31

**Authors:** Diyuan Yang, Jing Ning, Yuyu Zhang, Xuehua Xu, Dongwei Zhang, Huifeng Fan, Jing Wang, Gen Lu

**Affiliations:** a Department of Respiratory, Guangzhou Women and Children's Medical Center, Guangzhou Medical University, No. 9 Jinsui Road, Guangzhou, 510623, China; b Department of Pediatric Respiratory, Guangzhou women and children's medical center liuzhou hospital, Guangxi, Liuzhou, 545006, China; c Department of Children's Health Care, Guangdong Women and Children Hospital, Guangzhou Medical University, Guangzhou, 511442, China; d Department of Children's Health Care, Guangzhou Women and Children's Medical Center, Guangzhou Medical University, Guangzhou, 510623, China

**Keywords:** Adenovirus, Artemisinin, Artesunate, Artemisone, Antiviral activity, Viral replication, Drug repurposing

## Abstract

Adenoviral infections, particularly in children, remain a significant public health issue with no approved targeted treatments. Artemisinin and its derivatives, well-known for their use in malaria treatment, have shown antiviral activities in recent studies. However, their efficacy against human adenovirus (HAdV) remains unexplored. This study aimed to assess the activity of artemisinin and its derivatives against HAdV infection in vitro using cell lines and primary cells. Our data revealed that artemisinin exhibited dose-dependent anti-HAdV activity with no apparent cytotoxicity over a wide concentration range. Mechanistically, artemisinin did not affect viral attachment or entry into target cells, nor the viral genome entry into cell nucleus. Instead, it inhibited HAdV through suppression of viral DNA replication. Comparative analysis with its derivatives, artesunate and artemisone, showed distinct cytotoxicity and anti-adenoviral profiles, with artemisone showing superior efficacy and lower toxicity. Further validation using a primary airway epithelial cell model confirmed the anti-adenoviral activity of both artemisinin and artemisone against different virus strains. Together, our findings suggest that artemisinin and its derivatives may be promising candidates for anti-HAdV treatment.

## Highlights

• Artemisinin and derivatives show anti-HAdV activity with low cytotoxicity.
• Artemisinin inhibits HAdV by suppressing viral DNA replication.
• Artemisone demonstrates superior efficacy and lower toxicity.

## 1 Introduction

Human adenoviruses (HAdVs) represent a diverse group of pathogens known to cause a wide range of clinical manifestations, ranging from mild respiratory and gastrointestinal illnesses to severe systemic infections, particularly in immunocompromised individuals and pediatric populations (Shieh, 2022; Probst et al., 2021). Over the years, adenoviruses continue to pose significant public health challenges worldwide (Probst et al., 2021; Umuhoza et al., 2021).

Despite the considerable medical burden associated with adenovirus infections, effective antiviral therapies targeting these pathogens remain elusive (Lynch III and Kajon, 2021; Londeree et al., 2020; Georgi et al., 2020). Current treatment options primarily rely on supportive care, highlighting an urgent need for the development of novel therapeutic strategies (Hiwarkar et al., 2017; Florescu and Stohs, 2019). One promising avenue involves the exploration of "drug repurposing," leveraging existing drugs for new therapeutic indications, which offers a cost-effective and expedited approach to drug development (Pushpakom et al., 2019; Singh et al., 2020; Cha et al., 2018). The repurposing of existing approved drugs for HAdVs has also been tested recently, with some showing promising preclinical results (Saha and Parks, 2020; Dodge et al., 2021).

Artemisinin, a sesquiterpene lactone isolated from the sweet wormwood plant (*Artemisia annua*), has garnered substantial attention in recent years due to their potent antimalarial properties (Maude et al., 2010). Initially utilized as frontline treatments against malaria, artemisinin-based combination therapies (ACTs) have revolutionized the management of Plasmodium falciparum malaria and significantly contributed to global malaria control efforts (Nosten and White, 2007).

Over the years, many artemisinin derivatives have been made and tested to improve the performance of artemisinin in the treatment of malaria and also other diseases. Beyond their antimalarial activity, accumulating evidence suggests that artemisinin and its derivatives possess broad-spectrum antiviral properties against various viral pathogens, including herpes simplex virus (HSV), hepatitis B virus (HBV), hepatitis C virus (HCV), HIV-1 and SARS-CoV2 (Efferth, 2018; Efferth et al., 2008; D'Alessandro et al., 2020; Cao et al., 2020). However, the potential efficacy against adenovirus infections of artemisinin and its derivatives remains relatively unexplored.

This study aims to address this knowledge gap by investigating the in vitro anti-adenoviral activity of artemisinin, and its 2 derivatives, artesunate, and artemisone. By employing cell line and primary respiratory cell models, we seek to evaluate their therapeutic potential in combating adenovirus infections, and also to elucidate the possible mechanism underlying their antiviral effects. The findings from this research may offer valuable insights into the development of novel antiviral agents and inform future therapeutic strategies for the management of adenovirus-related diseases.

## 2 Methods

### 2.1 Compounds and cells

Artemisinin (CAS No.: 63,968–64–9; purity: 99.83 %) and artesunate (CAS No.: 88,495–63–0; purity: 99.56 %) were purchased from Selleckchem, and artemisone (CAS No.: 255,730–18–8; purity: >= 98.0 %) was purchased from MedChemExpress. All three compounds were first dissolved in DMSO at 200 mM as stocks, aliquoted and stored at – 20 °C. The stocks were then further diluted in complete cell culture medium at desired concentrations before use.

All cell lines used in the current study were purchased from the American Type Culture Collection (ATCC) and cultured according to the ATCC's suggestions. In brief, human lung epithelial cell line A549 and human cervical epithelial cell line HeLa were cultured in DMEM (GenomBio) supplemented with 10 % FBS (Hyclone) and antibiotics (GenomBio), and human lung epithelial cell line BEAS-2B was cultured in BEGM™ Bronchial Epithelial Growth Medium (Lonza). Primary human bronchial epithelial cells were purchased from PromoCell and cultured in Airway Epithelial Cell Growth Medium (PromoCell). All cell cultures were maintained in a 37 °C incubator with 5 % CO2.

### 2.2 Virus propagation and titration

Two HAdV strains, HAdV 3 and 7, were used in this study, and both were obtained from ATCC and propagated according to ATCC's suggestions. A549 and HeLa cells were used for HAdV3 and 7 propagations, respectively. Cells were first grown in T175 flasks to reach 70–85 % confluency and then viruses were inoculated at 0.1 MOI. After 3–4 days of culture, viruses were harvested and purified by a two-step CsCl gradient centrifugation, as previously described with modifications (Kanegae et al., 1994; Li et al., 2023). In brief, viruses were first released from infected cells by 3 freeze-thaw cycles, and then cell debris was removed by centrifugation at 10,000 g for 10 min. Cleared supernatants were subsequently loaded onto a 2.2 and 4 M CsCl discontinuous gradient and centrifuged at 25,000 rpm for 2 h (SW28, Beckman Coulter). The virus band was harvested, mixed with saturated CsCl at 1:1 ratio and overlaid with another 2.2 and 4 M CsCl discontinuous gradient, and centrifuged at 35,000 rpm for another 3 h (SW41Ti, Beckman Coulter). All centrifugations were done at 4 °C. Purified viruses were buffer exchanged into PBS + 10 % glycerol using the Amicon ultracentrifuge columns (Merck) and aliquoted and stored at −80 °C. To check virus titration, stored virus aliquots were thawed in 37 °C and then 10-fold serially diluted with complete cell culture medium. After dilution, the virus samples were subsequently added to target cell monolayers in 96 well plates and cultured for 48 h. Following incubation, cells were washed with PBS and fixed with cold methanol. HAdV-positive cells were then detected by an Adenovirus DFA Kit (EMD Millipore), according to the manufacturer's instructions. In brief, the culture medium was removed after incubation, and cells were washed with cold PBS and fixed with ice-cold 100 % methanol for 10 min at −20 °C. After fixation, cells were washed with PBST (PBS + 0.05 % Tween 20) and stained with Light Diagnostics Adenovirus DFA reagent for 30 min at 37 °C. Following staining, cells were washed again with PBST, mounted with Aqueous Mounting Medium (BosterBio), and the fluorescein isothiocyanate (FITC) signal was counted under a fluorescent microscope. The virus titer was then calculated as fluorescent-forming units (FFUs).

### 2.3 Cytotoxicity assay

The cytotoxicity of artemisinin, artesunate and artemisone was assessed by LDH assay with a commercial LDH—Cytox Assay Kit (BioLegend) and an MTT assay with a commercial MTT Cell Proliferation and Cytotoxicity Assay Kit (Beyotime), according to the manufacturers’ instructions. For the LDH assay, artemisinin, artesunate and artemisone were first serially diluted with complete cell culture medium and then added to cells preseeded in 96 well plates and cultured for 72 h. After incubation, LDH working solution was added to each well and incubated for 30 min at room temperature in the dark, followed by the addition of stop solution. The plate was then read at 490 nm for absorbance and cytotoxicity was calculated. To assess the solvent effect on cell viability, the highest DMSO concentration used for drug dilutions was included in the control wells without drug treatment. Cells treated with Lysis Buffer (from the assay kit) was used as positive control, which was considered as 100 % cell death. For the MTT assay, cells were treated the same way as the LDH assay for 72 h. After treatment, 10 µL of MTT solution was added into each well and the plate was incubated at 37 °C for 4 h. Following incubation, 100 µL of MTT dissolving solution was added into each well and further incubated at 37 °C until the formazan crystals were completely dissolved. The plate was then read at 570 nm to measure absorbance and calculate cell viability. To assess the solvent effect on cell viability, control wells containing the highest DMSO concentration used in drug dilutions were included without drug treatment. These control wells were considered 100 % viable for cell viability calculations.

### 2.4 Inhibition assay

Viral inhibition assay was done according to a previously established protocol with some modifications (Li et al., 2023). In brief, serially diluted artemisinin and its derivatives were first incubated with HAdV for 1 h at 37 °C, and then the mixture was added to A549 or BEAS-2B cells preseeded in 96 well plates and cultured for an additional 24 h. Following the incubation, cells were washed with PBS and fixed with cold 100 % methanol. HAdV-infected cells were detected using the Adenovirus DFA Kit (EMD Millipore), as detailed above in Virus propagation and titration subsection. To assess the solvent effect on cell viability, the highest DMSO concentration used for drug dilutions was included in the control wells without drug treatment. The IC50s were then calculated using cells treated with virus only being considered as 100 % infected.

### 2.5 Viral binding assay

Viral binding assay was done according to a previously reported protocol with some modifications (Li et al., 2023). In brief, A549 cells were first incubated with HAdV (200 MOI) in the presence or absence of artemisinin for 1 h at 4 °C (no virus-drug preincubation before cell treatment), and then cells were washed with cold sterile PBS to remove unbound virus. Following washes, cells were harvested, and total DNA was extracted using a commercial Magnetic Pathogen DNA Kit (Vazyme), according to the manufacturer's instructions. The viral DNA was then quantified by a SYBR-Green based real time PCR (RT-PCR), using previously defined conditions (Lee et al., 2005; Yu et al., 2021). The relative HAdV titer in the samples were calculated with the 2^−ΔΔCt^ method using GAPDH as the internal normalization control. The following primers were used for HAdV detection: nehex3deg (5′-GCCCGYGCMACNGANACSTACTTC-3′) and nehex4deg (5′-CCYACRGCCAGNGTRWANCGMRCYTTGTA-3′). These primers bind to a conserved region of the hexon gene across different HAdV subtypes. The following primers were used for the GAPDH internal control: GAPHD-F: (5′-GAGTCAACGGATTTGGTCGT-3′) and GAPDH-R (5′-TTGATTTTGGAGGGATCTCG-3′). The following PCR protocol was used: initial denaturation at 98 °C for 3 min, and 40 cycles of 98 °C, 30 s and 60 °C, 30 s.

### 2.6 Virus internalization assay

Virus internalization assay was done according to previously reported protocols with some modifications (Li et al., 2023; Wang et al., 1998). In brief, A549 cells were first incubated with HAdV (200 MOI) in the presence or absence of artemisinin for 1 h at 37 °C to allow viral infection and entry of target cells (no virus-drug preincubation before cell treatment). After incubation, cells were immediately transferred onto ice to pause the reaction. After removal of virus-containing medium, the cells were washed with PBS and unbound and cell surface viruses were removed by 0.25 % trypsin-EDTA solution at 37 °C for 5 min followed by PBS washes. Total DNA was then isolated from washed cells and HAdV DNA was quantified using the same RT-PCR as described in the viral binding assay.

### 2.7 Viral nuclear entry assay

Viral nuclear entry assay was done according to a previously reported protocol with modifications (Schreiner et al., 2012). In brief, A549 cells were first treated with HAdV in the presence or absence of artemisinin for 1 h at 37 °C (no virus-drug preincubation before cell treatment), and then unbound and cell surface viruses were removed by 0.25 % trypsin-EDTA solution followed by washes with cold PBS. Following washes, nuclei were isolated from pelleted cells by a commercial cytoplasm and nucleus isolation kit (ProteinTech), and nuclear total DNA was extracted using a commercial magnetic pathogen DNA kit (Vazyme), both according to the manufacturers’ instructions. In brief, cells were washed with cold PBS supplemented with phosphatase inhibitors (ProteinTech Kit) and pelleted at 300 g for 5 min at 4 °C. The cell pellet was resuspended in Hypotonic Buffer (ProteinTech Kit) and incubated on ice for 15 min to allow cells to swell. Detergent (ProteinTech Kit) was then added and mixed by vortexing for 10 s at the highest setting. Samples were centrifuged at 14,000 g for 30 min at 4 °C to pellet the cell nuclei. The nuclei pellets were washed with cold PBS, re-pelleted by centrifugation, and resuspended in cold PBS for nucleic DNA extraction. Nuclei were lysed with a mix of Proteinase K, Lysis Buffer 2, and Binding Buffer 2 (all from the Vazyme Magnetic Pathogen DNA Kit), and incubated with magnetic beads (Vazyme Magnetic Pathogen DNA Kit) to capture DNA. Following sequential washes with Wash Buffer A and B, the purified nucleic DNA was eluted into nuclease-free water. The relative HAdV titer in the isolated nuclear DNA was quantified using the same RT-PCR as described in the viral binding assay.

### 2.8 Viral replication assay

Viral replication assay was done according to a previously established protocol with modifications (Marrugal-Lorenzo et al., 2018). In brief, cells were first infected with HAdV in the presence or absence of artemisinin for 24 - 48 h at 37 °C (no virus-drug preincubation before cell treatment), and then cells were harvested, and total DNA was extracted using a commercial DNA isolation kit (Vazyme), according to the manufacturer's instructions. In brief, cells were lysed with a mix of Proteinase K, Lysis Buffer 2, and Binding Buffer 2 (all from the Vazyme Magnetic Pathogen DNA Kit), and incubated with magnetic beads (Vazyme Magnetic Pathogen DNA Kit) to capture DNA. Following sequential washes with Wash Buffer A and B, the purified nucleic DNA was eluted into nuclease-free water. HAdV DNA was then quantified using the same RT-PCR as described in the viral binding assay.

### 2.9 Viral early gene expression

Viral early gene E1A expression was quantified by RT-PCR, as previously described with modifications (Zhao et al., 2007). In brief, A549 cells were first infected with HAdV in the presence or absence of artemisinin, in the same way as in the viral replication assay, for 16 h at 37 °C. Then, total RNA was extracted using a VeZol-Pure Total RNA Isolation Kit (Vazyme), and E1A RNA level was quantified using a HiScript II One Step qRT-PCR SYBR Green Kit (Vazyme), both according to the manufacturer's instructions. In brief, cells were first washed with PBS and then lysed with VeZol Reagent for 5 min at room temperature. The lysate was mixed with 1/5 vol of chloroform, incubated for 5 min at room temperature, and centrifuged at 14,000 g for 5 min at 4 °C. The RNA-containing aqueous phase was transferred to a new tube, mixed with ½ volume of absolute ethanol, and loaded onto FastPure RNA Columns VI (Vazyme). Following sequential washes with Buffer RWA and RWB (Vazyme), RNA was eluted with nuclease-free water. The purified RNA was then used for one-step RT-PCR with the HiScript II One Step qRT-PCR SYBR Green Kit (Vazyme). The PCR program was as follows: reverse transcription at 50 °C for 3 min; initial denaturation at 95 °C for 30 s; 40 cycles of 95 °C for 10 s and 60 °C for 30 s. The relative E1A RNA level was calculated with the 2^−ΔΔCt^ method using GAPDH as the internal normalization control. The following primer pairs were used: E1A-F: (5′- TAGAGATAGACGGGCCGGAG-3′) and E1R: (5′- AGTCCATTTCAGCTGCTCCC-3′); GAPHD-F: (5′-GAGTCAACGGATTTGGTCGT-3′) and GAPDH-R (5′-TTGATTTTGGAGGGATCTCG-3′).

### 2.10 Progeny virus titer measurement

A549 cells were first infected with HAdV in the presence or absence of artemisinin, in the same way as in the viral replication assay, for up to 72 h at 37 °C. At 48 and 72 h, samples were collected, and progeny virus titers were titrated as detailed above in Virus propagation and titration subsection.

### 2.11 Statistical analysis

All data were expressed as mean +/- standard deviation and all statistical comparisons were made using the Kruskal-Wallis test followed by Dunn's multiple comparisons with GraphPad Prism 10. The statistical significance was set at a p values <0.05.

## 3 Results

### 3.1 Cytotoxicity and anti-HAdV activity assessment of artemisinin in airway epithelial cells

**Fig. 1:**
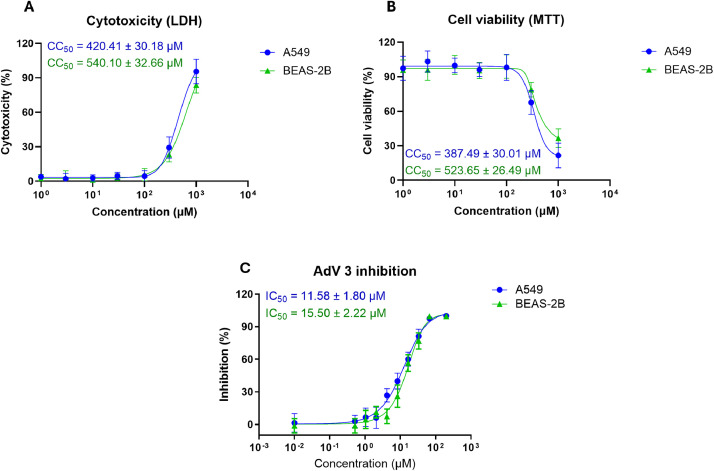
The cytotoxicity and anti-HAdV activity assessment of artemisinin on human airway epithelial cells. (A) Artemisinin was serially diluted and incubated with A549 and BEAS-2B cells for 72 h, and then cytotoxicity was measured by LDH assay and CC50 was calculated. (B) A549 and BEAS-2B cells were infected with HAdV3 in the presence of serially diluted artemisinin for 24 h and then viral infection was quantified and IC50 was calculated. Data shown are mean ± SD of 3 independent experiments.

The cytotoxicity of artemisinin to human airway epithelial cells and its activity against HAdV were first assessed. Two human airway epithelial cell lines, A549 and BEAS-2B, were used in this study to confirm that the results were not cell-line-dependent. Cytotoxicity was first assessed, and our data showed that artemisinin was not toxic when used at up to 100 µM on both human airway epithelial cell lines, and it started to rise in an artemisinin dose-dependent manner when used at concentrations higher than 100 µM (Fig. 1A-B). Next, the anti-HAdV activity of artemisinin was assessed in the non-cytotoxic concentration range. As shown in Fig. 1C and S1, artemisinin inhibited HAdV 3 infection in a dose-dependent manner, and such inhibition was observed in both A549 and BEAS-2B cells with comparable level of potency. In detail, the IC50 of artemisinin in A549 cells was 11.58 ± 1.80 µM while that in BEAS-2B cells was 15.50 ± 2.22 µM. Taken together, our data here indicate that artemisinin has anti-HAdV activity, and such activity was testing cell line-independent.

### 3.2 Artemisinin exerts anti-HAdV activity through inhibition on viral replication

**Fig. 2:**
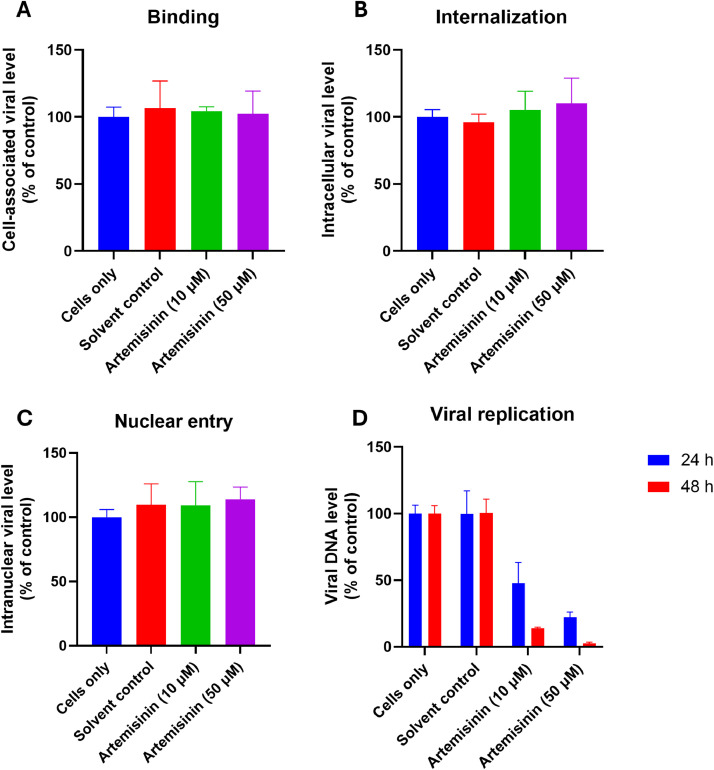
The impact of artemisinin on HAdV binding, internalization, viral genome nuclear entry and viral replication. (A) A549 cells were treated with HAdV3 in the presence or absence of artemisinin for 1 h at 4 °C, and then unbound viruses were washed and cell-associated viruses were quantified by PCR. (B) A549 cells were treated with HAdV3 in the presence or absence of artemisinin for 1 h at 37 °C, and then cell surface viruses were removed by 0.25 % trypsin-EDTA and internalized viruses were quantified by PCR. (C) A549 cells were first treated with HAdV3 in the presence or absence of artemisinin for 1 h at 37 °C, and then nuclei were harvested and viral DNA in the nucleus was quantified by PCR. (D) A549 cells were first treated with HAdV3 in the presence or absence of artemisinin for 24 - 48 h at 37 °C, and then cells were harvested and viral DNA was quantified by PCR. Data shown are mean ± SD of 3 independent experiments. The Solvent control group contained DMSO in the same concentration as the Artemisinin (50 µM) group. ns, not statistically significant; *, *p* < 0.05; **, *p* < 0.01.

We next further investigated the mechanism underlying the artemisinin induced HAdV inhibition. Successful HAdV infection can be divided into 4 steps: viral binding to target cell, virus internalization by target cell, viral genome entry of the infected cell nucleus and viral replication in the target cell. Our study aimed to examine which step in the HAdV infection cycle was impacted by artemisinin. Because the anti-HAdV activity of artemisinin was not cell line dependent, our next experiments were done in A549 cells only. The impact of artemisinin on virus binding to target cells was first evaluated. As shown in Fig. 2A, the presence of 10 or 50 µM artemisinin did not lead to apparent changes in cell-bound virus quantity, indicating that artemisinin does not affect viral attachment. Similarly, the internalization assay revealed that the viral genome copies were comparable between artemisinin-treated and untreated cells, indicating that this drug does not alter the virus internalization into the target cells (Fig. 2B). Quantification of nucleus-associated viral genomes confirmed that artemisinin did not seem to affect the nuclear entry of the HAdV3 genome, as the genome levels were comparable between treated and untreated cells (Fig. 2C). At last, the viral replication was assessed and we detected significant reduction in HAdV replication in the presence of artemisinin, and such reduction seemed to be artemisinin dose and treatment time-dependent (Fig. 2D). The inhibition of artemisinin on HAdV3 replication was stronger at 48 h post-infection than 24 h, indicating that the viral replication was lower at 24 h than at 48 h (Liu et al., 2020). Since viral replication relies on the expression of its early genes, we also checked whether the early gene E1A expression was affected by drug treatment and consequently contributed to the reduced viral replication. Our data showed that E1A RNA levels were not apparently altered by artemisinin treatment, indicating that artemisinin inhibits HAdV3 replication through an E1A-independent mechanism (Fig. S2). Further, we evaluated whether the decrease in virus replication caused by artemisinin treatment also led to reduced progeny virus production. Progeny virus titers were measured at 48 and 72 h post-infection. Our data showed that the artemisinin-treated groups had significantly reduced virus production in a dose- and time-dependent manner (Fig. S3). Taken together, our data here indicate that artemisinin inhibits HAdV infection by limiting viral replication in the target cells.

### 3.3 Artemisinin derivatives demonstrate varied levels of cytotoxicity to airway epithelial cells and anti-HAdV activity

**Fig. 3:**
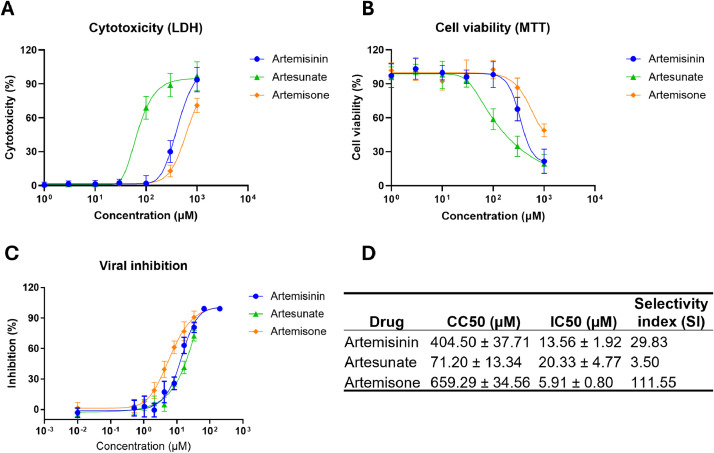
The cytotoxicity and anti-HAdV activity assessment of artemisinin derivatives on human airway epithelial cells. (A) Artemisinin and derivatives were serially diluted and incubated with A549 cells for 72 h, and then cytotoxicity was measured by LDH assay. (B) A549 cells were infected with HAdV3 in the presence of serially diluted artemisinin and derivatives for 24 h and then viral infection was quantified. Data shown are mean ± SD of 3 independent experiments. (C) CC50, IC50 and SI were calculated.

Since the first discovery of artemisinin in 1972, various derivatives have been developed with the purpose to improve water solubility and anti-malaria activity. We next compared the cytotoxicity and anti-HAdV activity of different artemisinin derivatives, in order to check if its derivatives also could show better efficacy against HAdV like they did in malaria. Two artemisinin derivatives, artesunate and artemisone, were tested in the current study. Artesunate was developed to address challenges associated with the rapid metabolism and poor water solubility of artemisinin, and artemisone was developed as a more potent and stable derivative artemisinin with improve anti-malaria properties (Zhang et al., 2022; Obaldia et al., 2009). Our data showed that artemisinin and its 2 derivatives all exhibited activity against HAdV, but they showed quite different cytotoxicity and anti-HAdV profiles (Fig. 3A-D and S4). In detail, using artemisinin as a reference (CC50 = 404.50 ± 2.22 µM; IC50 = 13.56 ± 1.92 µM), artesunate demonstrated higher cytotoxicity (CC50 = 71.20 ± 13.34 µM) but lower anti-HAdV activity (IC50 = 20.33 ± 4.77 µM), while artemisone exhibited lower cytotoxicity (CC50 = 659.29 ± 34.56 µM) and higher anti-HAdV activity (IC50 = 5.91 ± 0.80 µM). Selectivity indices (SI = CC50/IC50), a measure to assess the relative safety and efficacy of a drug, for these 3 drugs against HAdV were also calculated. Our data showed that artemisone was the best performer of 3, with SI = 111.55, followed by artemisinin (SI = 29.83), while artesunate was the last (SI = 3.50; Fig. 3D). Taken together, our data here indicate that artemisinin derivatives have varied levels of activities against HAdV, and of the 3 tested derivatives, artesunate is less effective than artemisinin while artemisone is superior to artemisinin.

### 3.4 The anti-HAdV activity assessment of artemisinin and derivative artemisone on a primary airway epithelial cell model

**Fig. 4:**
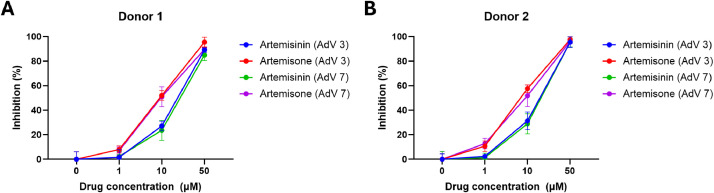
Anti-HAdV activity assessment of artemisinin and derivatives on primary human airway epithelial cell model. Human epithelial cells from (A) donor 1 and (B) donor 2 were first treated with HAdV3 or 7 in the presence or absence of artemisinin or artemisone for 24 h, and then virus infected cells were quantified and viral inhibition was calculated.

To further confirm our findings, we next assessed the anti-HAdV activities of artemisinin and its derivatives on a primary human airway epithelial cell model. Since artesunate was less effective than artemisinin in the previous test, we tested artemisinin and its derivative artemisone here. To further confirm the anti-HAdV activity of artemisinin and its derivative was effective only against HAdV 3, we included another common respiratory HAdV 7 in this experiment. As shown in Fig. 4A-B, both artemisinin and artemisone inhibited HAdV infection in the primary airway epithelial cell model, and in consistent with our previous results, artemisone still showed better activity than artemisinin. Furthermore, both drugs inhibited HAdV 7 infection to a similar level that they showed inhibition against HAdV 3, implying their anti-HAdV activity may not be HAdV strain restricted (Fig. 4A-B). Taken together, our data confirm on a primary airway epithelial cell model that artemisinin and its derivative artemisone can inhibit HAdV infection and such inhibition is likely to be effective against different HAdV strains.

## 4 Discussion

Artemisinin and its derivatives, initially developed for the treatment of malaria, have been found to possess significant antiviral and anticancer properties beyond their original purpose (Efferth, 2018; Efferth et al., 2008; D'Alessandro et al., 2020; Cao et al., 2020). Our study adds to this growing body of evidence by demonstrating the anti-HAdV activity of artemisinin and its derivatives. This discovery underscores the versatility of artemisinin-based compounds and their potential to be repurposed for treating various viral infections, thereby broadening their therapeutic applications. Our findings indicate that artemisinin exhibits minimal cytotoxicity to human airway epithelial cells while effectively inhibiting HAdV 3 infection in a dose-dependent manner, primarily through the suppression of viral replication.

Currently, there are no specific antiviral drugs approved for the treatment of adenovirus infections. The development of new antiviral drugs is typically a time-consuming and financially burdensome process, making it less attractive to pharmaceutical companies. Drug repurposing, which involves finding new therapeutic uses for existing drugs, offers a more cost-effective and expedited approach to drug development (Pushpakom et al., 2019). Our findings support the potential of artemisinin and its derivatives as viable candidates for repurposing in the fight against adenovirus infections, highlighting the importance of exploring this avenue further. This is particularly significant given the urgent need for effective adenovirus treatments, as current therapeutic options are limited to supportive care (Lynch III and Kajon, 2021).

Our study has shown that while artemisinin derivatives exhibit anti-HAdV activity, their efficacies vary. Our finding is in consistent with previous studies that artemisinin and its derivatives show varied levels of activity against CMV and SARS-CoV2 infection (Cao et al., 2020; Oiknine-Djian et al., 2018). Specifically, we found that artemisone demonstrated superior efficacy and lower cytotoxicity compared to artemisinin and artesunate. This variability suggests that further screening of artemisinin derivatives against HAdV is warranted to identify the most potent candidates. By systematically evaluating a broader range of derivatives, we may discover compounds with even greater efficacy, which could lead to more effective treatments for adenovirus infections. Such an approach could significantly enhance the therapeutic options available for managing these infections.

The three drugs tested in our study exhibited very different cytotoxicity profiles (close to 10-folds between artemisinin and artesunate), suggesting that the cells have varying tolerance levels towards these drugs. Since the artesunate is modified based on artemisinin, the additional chemical groups may contribute to increased toxicity in cells. However, the antiviral efficacy is comparable between these 2 drugs, indicating that the antiviral functional groups are not significantly enhanced or diminished by these chemical modifications.

Artemisinin, initially discovered as an antimalarial drug, and its derivatives have been shown to exhibit broad-spectrum antiviral and anticancer activities, though the mechanisms underlying their actions against different pathogens remain debated or elusive. The exact mechanism of artemisinin's antimalarial activity is still under debate, with several proposed mechanisms, including interference with hematin formation and heme accumulation (Robert et al., 2005; Bousejra-El Garah et al., 2008), covalent reaction with parasitic proteins causing protein alkylation (Meshnick et al., 1994; Ying-Zi et al., 1993; Wu et al., 2003), inhibition of the parasite's PfATP6ase (an enzyme orthologous to sarco/endoplasmic reticulum membrane calcium ATPase) (Eckstein-Ludwig et al., 2003), and accumulation within neutral lipids causing parasite membrane damage (Hartwig et al., 2009). Regarding its anticancer activity, it remains unclear whether definitive molecular targets are involved, but it is suggested that translationally controlled tumor protein (TCTP) may be a target, as tumor cells with high TCTP expression are sensitive to artesunate, while those with low TCTP expression show resistance (Efferth, 2005). Additionally, artesunate can act as a topoisomerase inhibitor, blocking the ligation step of the cell cycle (Youns et al., 2009). The antiviral mechanisms of artemisinin are not yet extensively studied, but several have been proposed for different viruses: in HCMV, artesunate inhibits viral replication through direct or indirect interactions with NF-κB or Sp1 (Eickhoff et al., 2004; Efferth et al., 2002), and in flaviviruses, artemisinin-induced antiviral activity is associated with an enhanced type I interferon response (Wang et al., 2020). Our study has shown that artemisinin and its derivatives exhibit anti-HAdV activity, warranting future studies to explore whether these drugs function through the currently identified pathways or other yet-to-be-discovered mechanisms.

Although artemisinin and its derivatives are repurposed drugs that have undergone extensive safety testing in clinical settings for their antimalarial use, their anti-adenoviral activity requires further investigation. Comprehensive preclinical and clinical evaluations are necessary to fully understand their efficacy, safety, and mechanisms of action against adenovirus infections. Such studies will be crucial in establishing the clinical relevance of artemisinin and its derivatives for treating adenovirus-related diseases and ensuring their safe and effective use in patients.

## 5 Conclusions

Our study demonstrates that artemisinin exhibits minimal cytotoxicity to human airway epithelial cells while effectively inhibiting HAdV 3 infection in a dose-dependent manner, primarily through the suppression of viral replication. Comparison with its derivatives, artesunate and artemisone, reveals distinct cytotoxicity and anti-HAdV profiles, with artemisone showing superior efficacy and lower toxicity. Furthermore, validation on a primary airway epithelial cell model confirms the anti-HAdV activity of both artemisinin and artemisone against different HAdV strains. These findings highlight the potential of artemisinin-based compounds, particularly artemisone, as promising therapeutic agents for the treatment of adenovirus infections, warranting further investigation into their clinical application.

## Funding sources

This work was supported by the self-raised research funds in Western medicine in Guangxi [Z-B20241308], the Science and Technology Program of Guangzhou [202102080493], and the Foundation of Guangdong Administration Bureau of Chinese Medicine [20231264].

## CRediT authorship contribution statement

**Diyuan Yang:** Writing – original draft, Methodology, Investigation, Formal analysis, Data curation, Conceptualization. **Jing Ning:** Writing – review & editing, Visualization, Methodology, Formal analysis, Data curation, Conceptualization. **Yuyu Zhang:** Writing – review & editing, Validation, Formal analysis, Data curation. **Xuehua Xu:** Writing – review & editing, Validation, Data curation. **Dongwei Zhang:** Writing – review & editing, Data curation. **Huifeng Fan:** Writing – review & editing, Data curation. **Jing Wang:** Writing – review & editing, Funding acquisition, Formal analysis. **Gen Lu:** Writing – review & editing, Writing – original draft, Supervision, Conceptualization.

## Declaration of competing interest

The authors declare that they have no known competing financial interests or personal relationships that could have appeared to influence the work reported in this paper.

## Data Availability

The data that support the findings of this study are available from the corresponding author upon reasonable request.

## Appendix Supplementary materials

Image, application 1
