# Supplementary material for: In vitro assessment of the anti-adenoviral activity of artemisinin and its derivatives
Source: Virus Res. 2024 Aug 31;349:199448. doi: 10.1016/j.virusres.2024.199448 (PMC11403056; doi:10.1016/j.virusres.2024.199448)
Supplement: Supplementary file 1 [file mmc1.docx]

Supplementary materials

**Figure S1.** The anti-HAdV activity assessment of artemisinin on human airway epithelial cells. A549 and BEAS-2B cells were infected with HAdV3 in the presence of serially diluted artemisinin for 24 h and then viral infection was quantified. Data shown are mean ± SD of 3 independent experiments.

**Figure S2.** The impact of artemisinin on HAdV early gene E1A expression. A549 cells were first treated with HAdV3 in the presence or absence of artemisinin for 16 h at 37 °C, and then cells were harvested and viral DNA was quantified by PCR. Data shown are mean ± SD of 3 independent experiments. The Solvent control group contained DMSO in the same concentration as the Artemisinin (50 µM) group.

**Figure S3.** The impact of artemisinin on HAdV progeny virus titer. A549 cells were first treated with HAdV3 in the presence or absence of artemisinin for 48 – 72 h at 37 °C, and then samples are harvested at 48 h and 72 h and progeny virus titers were measured. Data shown are mean ± SD of 3 independent experiments. The Solvent control group contained DMSO in the same concentration as the Artemisinin (50 µM) group.

**Figure S4.** The anti-HAdV activity assessment of artemisinin derivatives on human airway epithelial cells. A549 cells were infected with HAdV3 in the presence of serially diluted artemisinin and derivatives for 24 h and then viral infection was quantified. Data shown are mean ± SD of 3 independent experiments.
